# Walking to your right music: a randomized controlled trial on the novel use of treadmill plus music in Parkinson’s disease

**DOI:** 10.1186/s12984-019-0533-9

**Published:** 2019-06-07

**Authors:** Rocco Salvatore Calabrò, Antonino Naro, Serena Filoni, Massimo Pullia, Luana Billeri, Provvidenza Tomasello, Simona Portaro, Giuseppe Di Lorenzo, Concetta Tomaino, Placido Bramanti

**Affiliations:** 1 IRCCS Centro Neurolesi Bonino Pulejo, via Palermo, Contrada Casazza, S.S. 113, 98124 Messina, Italy; 2 Fondazione Centri di Riabilitazione Padre Pio Onlus, San Giovanni Rotondo, FG Italy; 3 Institute for Music and Neurologic Function, Mount Vernon, NY USA

**Keywords:** Gait rehabilitation, Parkinson’s disease, Rhythmic auditory stimulation, GaitTrainer3

## Abstract

**Background:**

Rhythmic Auditory Stimulation (RAS) can compensate for the loss of automatic and rhythmic movements in patients with idiopathic Parkinson’s disease (PD). However, the neurophysiological mechanisms underlying the effects of RAS are still poorly understood. We aimed at identifying which mechanisms sustain gait improvement in a cohort of patients with PD who practiced RAS gait training.

**Methods:**

We enrolled 50 patients with PD who were randomly assigned to two different modalities of treadmill gait training using GaitTrainer3 with and without RAS (non_RAS) during an 8-week training program. We measured clinical, kinematic, and electrophysiological effects of both the gait trainings.

**Results:**

We found a greater improvement in Functional Gait Assessment (*p* < 0.001), Tinetti Falls Efficacy Scale (*p* < 0.001), Unified Parkinson Disease Rating Scale (*p* = 0.001), and overall gait quality index (*p* < 0.001) following RAS than non_RAS training. In addition, the RAS gait training induced a stronger EEG power increase within the sensorimotor rhythms related to specific periods of the gait cycle, and a greater improvement of fronto-centroparietal/temporal electrode connectivity than the non_RAS gait training.

**Conclusions:**

The findings of our study suggest that the usefulness of cueing strategies during gait training consists of a reshape of sensorimotor rhythms and fronto-centroparietal/temporal connectivity. Restoring the internal timing mechanisms that generate and control motor rhythmicity, thus improving gait performance, likely depends on a contribution of the cerebellum. Finally, identifying these mechanisms is crucial to create patient-tailored, RAS-based rehabilitative approaches in PD.

**Trial registration:**

NCT03434496. Registered 15 February 2018, retrospectively registered.

## Introduction

The loss of automaticity and rhythmicity of movements in patients with idiopathic Parkinson’s disease (PD) has been correlated with the presence of different gait abnormalities, including shuffling steps, gait initiation failure, and freezing of gait [33], which all make the gait rehabilitation challenging in these patients [12]. The loss of automaticity and rhythmicity may depend on the impairment of the cerebral mechanisms that generate a regular walking rhythm [28], possibly because of deficient dopamine levels within the cortical-striatal locomotor network [23, 24, 60]. Indeed, humans synchronize their movements with external rhythmic cues through an innate internal timing process (i.e., rhythmic entrainment) [81]. This process involves different frontoparietal networks, including auditory, premotor, and motor areas [4, 8, 37], which are connected across complex basal ganglia(BG)-thalamo-cortical and cerebello-thalamo-cortical motor networks, as suggested by some connectivity studies showing abnormalities in neural activity and connectivity within frontoparietal networks in patients with PD. [4, 27, 46, 62, 63, 76, 79]

Therefore, gait rehabilitation in patients with PD is aimed at restoring the cerebral mechanisms that generate a regular walking rhythm. These patients have been provided with a walking treadmill equipped with rhythmic auditory stimulation (RAS) to improve gait parameters by harnessing the innate internal timing process (i.e., rhythmic entrainment) through external cues [49, 51, 52, 59, 69, 81, 82, 85]. Treadmill walking by itself alone has been found to furnish lasting, positive effects on different gait parameters, probably by affecting specific neuroplasticity mechanisms within complex cortical-BG-cerebellar networks [64]. However, auditory cues significantly improve gait parameters [37], probably by providing an external rhythm that bypasses the internal rhythm deficit [49, 54] by engaging complex frontoparietal connections based on complex cortico-BG-cerebellar loops [9]. This could compensate for any failure in the mechanisms controlling automatic and rhythmic movement generation [54]. By coupling steps with external auditory cues, it could be possible to form a rhythmic gait by entraining movement patterns, i.e., via frequency locking between two oscillating bodies [49, 51, 52, 84] to support the generation of better gait patterns; the rhythmic entrainment; the engagement of automatic timing systems; the planning, performing, and learning of movements; the acquisition of temporal skills; and an increase in motivation [81–84].

This type of coupling has been shown to improve several gait parameters, including cadence, gait velocity, stride length [23, 44, 69, 77, 86], gait timing variability [14, 49, 51, 52, 89], the pedaling rate [21], and step amplitude ([1]). Moreover, it has been shown that other motor parameters, such as the Unified Parkinson Disease Rating Scale (UPDRS) scores and freezing of gait, as well as cognitive processes and motor learning processes also improve ([1, 11, 13, 35, 38, 39, 44, 49, 71, 59, 80]).

Nonetheless, the neurophysiological mechanisms by which coupling steps with external auditory cues improves gait remain partially unclear [4, 13, 37]. Obtaining a better understanding of these neurophysiological mechanisms would allow clinicians to tailor neurologic music therapy-based rehabilitative approaches to individual patient (i.e., adapt their approach to the underlying neurophysiological basis) to improve the patients’ ability to generate a regular walking rhythm [78].

Investigating changes (increase or decrease) in gait cycle-related, frequency-band specific electroencephalography (EEG) power (namely, event-related desynchronization (ERD) and synchronization (ERS)) [65, 66] and of gait cycle-related, frequency-band specific coherence (namely, task-related coherence -TRCoh) [19, 48] induced by RAS gait training could offer useful information. In fact, the former approach may furnish information on the ongoing activities related to the motor process characteristics coded into the sensorimotor areas, including its kinematics (speeds) and kinetics (motor loads) [17, 55]. The latter approach offers useful information regarding the sensorimotor events related to the dynamic coupling between different brain areas (including the frontal and sensorimotor regions) [18, 48] and is thus an indicator for the network activity related to gait cycle generation. Moreover, using EEG is advantageous for capturing gait cycle-related dynamics as this tool is applicable in a mobile setup and provides good temporal resolution with regard for the brain activity. Therefore, ERS, ERD, and TRCoh data could be important for analysing the recovery mechanisms related to post-stroke brain function recovery ([10, 91]).

The aim of our study was to evaluate the efficacy of treadmill gait training combined with RAS in terms of mobility, balance, and gait parameters by correlating EEG changes with behavioral (gait) changes to identify the putative neurophysiological basis underlying gait improvement. To this end, we evaluated α (8–12 Hz) and β (13–28 Hz) frequency range changes in power (as estimated by time-frequency analysis) and coherence (as estimated by TRCoh) within the frontal, centroparietal, and temporal areas induced by treadmill gait training (GaitTrainer3; Biodex, Shirley, NY, US) with and without RAS in a group of patients with PD. We focused our analysis on α and β rhythms because these are thought to be a marker of the progression of the disease, patients’ responses to physiotherapy (including gait), and the effects of levodopa on motor symptoms [6, 7, 18, 47, 70], and they thus offer potentially useful information concerning gait impairment and responsiveness to treatment in patients with PD.

## Methods

### Trial design

Patients were enrolled in a parallel-group, randomized clinical trial. Patients were randomly allocated into either the RAS treadmill group or the non_ RAS treadmill gait training group. Regardless of group allocation, all patients were provided with a daily training program consisting of 45 min of conventional overground gait training, 45 min of activities in daily living training and reaching activities in occupational therapy, 45 min of biomechanical training in both the upper and lower limbs, 30 min of speech therapy, and 30 min of rest distributed between the sessions (for a total of 195 min). Then, the individuals were provided with further 30 min of RAS or non_RAS treadmill time, depending on the group assignment. The daily training program was practiced once a day at the same time of day (from 9 am to 1 pm), five times per week for eight consecutive weeks. RAS and non_RAS treadmill sessions were performed individually in the same location and supervised by physiotherapists with a 2 years of training in RAS. Three to four patients were supervised by each RAS-trained physiotherapist throughout the training period. The subjects were in a clinically ON phase when provided with the training, as per the UPDRS.

### Participants

Fifty out of 67 of the in-patients attending the Robotic Neurorehabilitation Unit of our Institute with a diagnosis of idiopathic Parkinson’s Disease (according to the UK Brain Bank diagnostic criteria) were rated as eligible to be enrolled in this randomized, assessor-blinded, parallel-group study. The inclusion criteria were as follows: (i) Hoehn and Yahr stage between II and III, Mini-Mental State Examination test > 23, and normal executive function tests [2, 41, 43, 50]; and (ii) no changes in antiparkinsonian drug treatment in the previous 6 months. The exclusion criteria included a history of neoplasms; severe cardiovascular, respiratory, visual, auditory, and muscular-skeletal disease; other neurological conditions; and neurologic music therapy in the last 3 months. The clinical-demographic characteristics are reported in Table 1. This study was approved by our local Ethics Committee and retrospectively registered on 15 February 2018 in ClinicalTrials.gov under no. NCT03434496 (https://clinicaltrials.gov/ct2/show/NCT03434496 NCT03434496). All participants gave written informed consent to study participation and data publication before the enrollment.

**Table 1:** Baseline parameters

Group	Age (yy ± s.d.)	Gender F/M	dd (yy ± s.d.)	H&Y (m ± s.d.)	MMSE (m ± s.d.)	CoM	Levodopa (mg ± s.d.)
RAS (*n* = 25)	70 ± 8	9/11	10 ± 3	3 ± 1	26 ± 3	None:4, DM:4, h:7, d:4, t:5, a:1	450 ± 55
non_RAS (*n* = 25)	73 ± 8	6/14	9.3 ± 3	3 ± 1	25 ± 3	None:5, DM:4, h:6, d:3, t:6, a:1	435 ± 49
*p*-value	0.7	0.4	0.3	0.1	0.2	0.7	0.7

RAS Rhythmic auditory stimulation; dd disease duration; MMSE Mini-Mental State Examination; H&Y Hoehn and Yahr; CoM comorbidities (DM diabetes mellitus, h blood hypertension, d dyslipidemia, t tabagism, a alcoholism)

### Intervention

GaitTrainer3 is a platform that integrates gait training via a treadmill and RAS. The device is indeed equipped with an instrumented deck that issues acoustic cues to determine the exact tempo and rhythm during gait training and visual real-time biofeedback to prompt patients to follow their gait pattern. In fact, the device provides online feedback, including step length, speed, and symmetry, to encourage patient progress and monitor patient performance. Patient footfalls were compared in real-time to the desired footfalls step by step and documented in a histogram.

Patients were required to walk along with the music “angel elsewhere”, which reaches a target music tempo of ~ 120 bpm. The song was presented with the lyrics, and the beat of the song was emphasized with a superimposed salient high-pitch bell sound. The patients were first trained to synchronize their footsteps to the beat of the music, which was adapted to their baseline gait performance; that is, the beat frequency of the RAS (namely, the beat rate of the music) was individually adjusted for each patient starting from the patient’s best cadence (gait frequency and stride length). Then, the beat frequency was progressively increased up to the target beat frequency (120 bpm) through the first three to five sessions. This frequency was then implemented for the remaining part of the RAS training. We adopted this intermediate target frequency and RAS setup as it has been shown that using a beat frequency not based on the patient’s baseline cadence can worsen step length and gait cadence, especially when the frequency is set too low (60-90 bpm) or too high (> 150 bpm) [42]. Moreover, RAS tasks that are not provided to the patient with the explicit instruction to synchronize their walking pace with the beat when adopting freely chosen music (i.e., not controlled for meter, rhythm or rate) or when combined with other cues (e.g., tactile stimuli) can negatively affect gait performance, perhaps because their attention is diverted to additional tasks irrelevant to walking [42].

### Outcomes

**Fig. 1:**
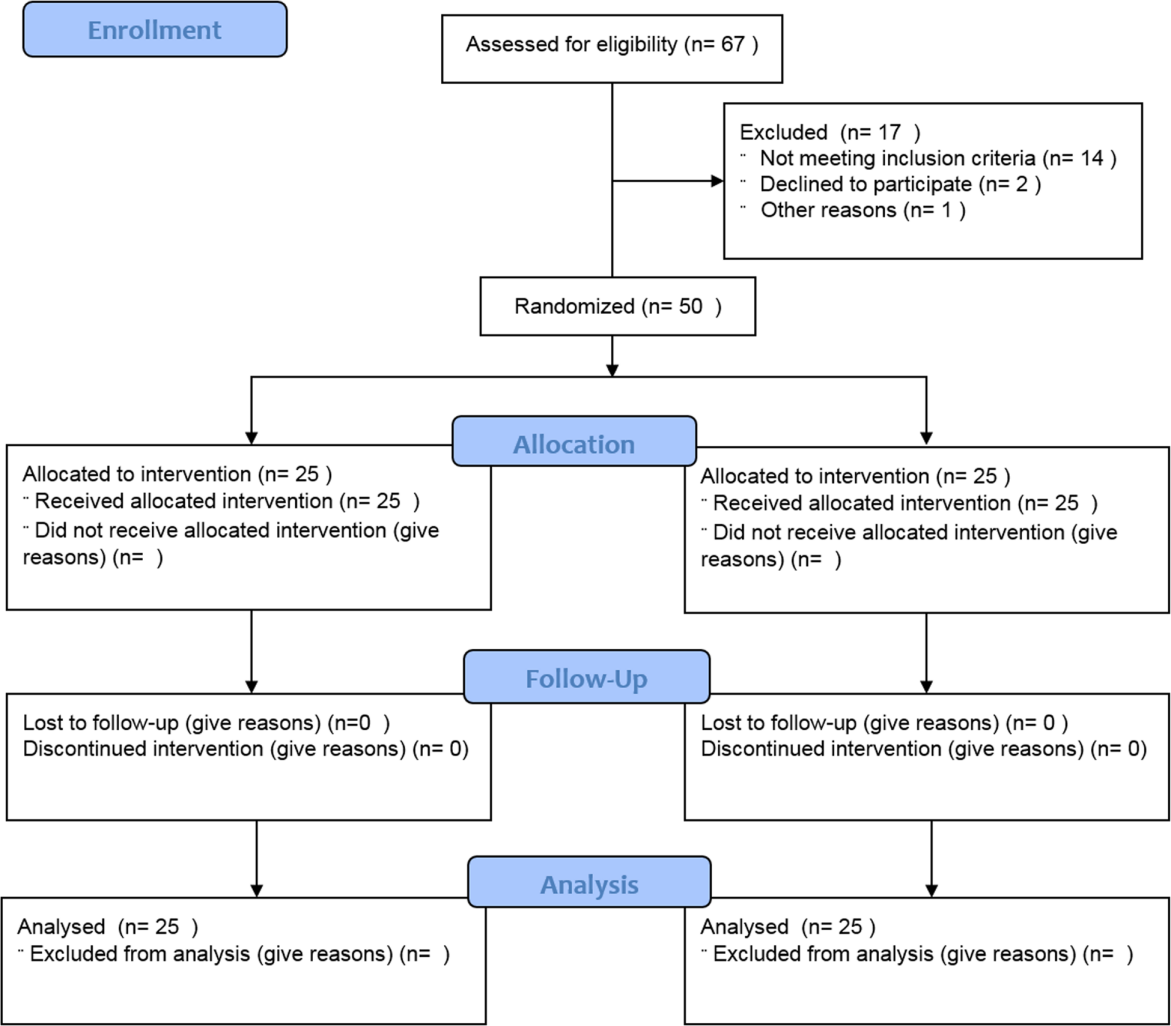
Summary of patients’ flow

Outcome measures were assessed before (TPRE) and after (TPOST) rehabilitation training was complete. The primary endpoint with respect to the clinical efficacy of gait training was the achievement of the minimal clinically important difference (MCID) in the Functional Gait Assessment (FGA) (at least 4 points). As secondary outcomes, we assessed the brain oscillation changes related to gait cycle (α and ERS/D magnitude changes) recorded by the frontal, centroparietal, and temporal pooled electrodes and the α and β TRCoh recorded by electrode-group pairs, which have been proposed to be correlated with the progression of the disease, the response to physiotherapy, and levodopa administration ([6, 7, 18]); they therefore offer potentially useful information concerning gait impairment and responsiveness to treatment in patients with PD. Furthermore, we calculated the results of the UPDRS, the Berg Balance Scale (BBS), the Tinetti Falls Efficacy Scale (FES), the 10-m walking test (10MWT), the timed up-and-go test (TUG), and the gait quality index (GQI) derived from a gait analysis sensor. During the 8-week training period, the patients were asked not to undertake other gait training regimens. The experimenters and those who analyzed the data (different from the first experimenters) were blind to patient allocation. The patient flow procedure is summarized in Fig. 1.

### EEG recording and analysis

Brain activity (EEG; μV) was continuously recorded for 10 min while the patient was walking on the GaitTrainer3 in the non_RAS modality, usually 5–10 min after the session started. We used a Brain-Quick System (Micromed; Mogliano Veneto, Italy) equipped with a standard 19-electrode headset. EEG recording occurred during the third to fifth session (depending on when a target gait of 120 bpm was reached) and the last gait training session. Patients were prohibited from drinking coffee, smoking, and changing their bedtime during the 3 days prior to EEG recording. This was easily checked, as the participants were in-patients.

EEG were sampled at 512 Hz, band-pass filtered between 1 and 200 Hz using a zero-phase finite impulse response (FIR) filter (order = 7500) to minimize drifts and a zero-phase FIR filter order = 36, referenced to Cz, and notch-filtered at 50 Hz (FIR notch filter, order = 3302) to remove the power line noise. Impedances were constantly kept below 5 kΩ for the entire duration of the experiment and data collection. An electro-oculogram (EOG) with a bipolar montage was also collected.

Data were pre-processed using EEGLab. EEG recordings were first visually inspected to identify and remove data affected by prominent artefacts across all the recording channels. Then, the data were re-filtered between 8 and 40 Hz, re-referenced to the common average reference, and decomposed into neural and artifactual components using the Infomax algorithm Independent Component Analysis (ICA) [15].

Continuous data were then segmented into epochs starting from the right heel strike (HS) and ending at the next one to capture a complete stride (composed of the following, in order: right, left, and right HS), to obtain 428 ± 25 epochs. EEG segmentation was based on data synchronized from the important time points (i.e., start, heel strikes, and end) furnished by a wireless inertial sensor (GSensor, BTS Bioengineering; Milan, Italy) and used to extrapolate gait phase data. Thus, the single trial spectograms were time-warped over the trials using a linear interpolation function, with the gait data used as milestones for realigning the EEG signals’ time axes (i.e., aligning the time-points of the epochs for the HS, including the right, left, and right HS time-warped to 0, 50, and 100% of the gait cycle, respectively) [25, 29, 67, 72, 73, 88].

To assess whether the RAS-induced changes in gait performance resulted in ERS/ERD strength variations in the α and β frequency range, we performed a time–frequency analysis related to the phases of the gait cycle [40]. To calculate ERS/ERD as a function of time, we employed a sinusoidal wavelet transform in which the data window length depended inversely on the frequency to obtain a better compromise between time changes and frequency changes [75]. ERS/ERD was defined as a percentage of power decrease in a specific frequency band relative to the baseline period.

The time-frequency coherence (i.e., the relationship between two non-stationary processes) was computed in terms of TRCoh to investigate inter-regional connectivity during gait (that is, the oscillatory aspects of interregional brain activation). TRCoh refers to the steady-state changes in functional connectivity associated with continuous tasks, that is, the ongoing sequential movements of the lower limbs rather than the phasic changes associated with single limb movements across the gait cycle, as is done to compute ERS/ERD. Furthermore, TRCoh approach eliminates the coherences that are not task-related (e.g., are due to volume conduction or controlled by the reference, thus equally present during both activation and rest conditions) [19, 48, 68]. Specifically, we computed the coherence for all possible pooled-electrode pairs for the α and β bands. Coherence values were calculated for each frequency bin as a complex correlation coefficient based on the value of the cross-spectrum for the pooled-electrode pair for a given frequency bin and the values of the autospectra for each electrode pool of the pair. Based on these values, coherence was obtained by squaring the magnitude of the complex correlation coefficient (ranging from 0 and 1). Consequently, coherences for each frequency bin were summed and divided by the number of frequency bins. Finally, TRCoh was obtained by subtracting the coherence values obtained during rest from those obtained during the corresponding activation conditions. Therefore, positive values indicated TRCoh magnitude increments, whereas negative values indicated TRCoh magnitude decrements.

### Gait data analysis

A single wireless inertial sensor (GSensor, BTS Bioengineering; Milan, Italy) was fixed to the subject’s waist with a semi-elastic belt to cover the L4–L5 inter-vertebral space. Gait data were continuously recorded for 30 s while the patient was walking on the GaitTrainer3 in the non_RAS mode at an individually adapted step cadence simultaneously with EEG recording. Recording occurred two successive times for 30 s each. The sensor provided acceleration data along the antero-posterior, medio-lateral and superior-inferior orthogonal axes, which were transmitted to a PC via Bluetooth and analyzed using dedicated software (BTS G-STUDIO). This software analysis furnished the gait phase data, including the speed of gait, step cadence, stride length, gait cycle duration, stance/swing ratio, and the GQI (an overall gait performance score reflecting the grand-average of the gait parameter with an approximate 60:40% distribution of stance:swing phases). All of the parameters were measured before and after gait training.

### Sample size

For the power analysis, we considered the effect of the RAS on FGA as the primary outcome measure at the end of the rehabilitation period. The FGA is a validated measurement of gait-related activities, balance, and gait ability and has been shown to have good construct validity in patients with PD, to have moderate-to-strong correlation with other balance and gait appraisals, and to predict falls within the subsequent 6 months [90]. We had to modify the outcomes of the protocol (as originally registered) before starting patient recruitment as we found that the pre-planned endpoints were not sufficient for our purposes according to the evidence coming from former trials and reviews. According to our experience and data in the literature [5], the required sample size was 25 patients per arm to detect a pre- to post-treatment MCID in the composite primary outcome. (i.e., a difference of at least 4 points with a standard deviation between 20 and 25% for each group, a two-sided confidence interval 95% and a power of 80% with a possible drop-out rate of10%) [3].

### Randomization and blinding

Patients were randomly allocated into either the RAS treadmill or the non_RAS treadmill gait training group at a 1:1 allocation ratio. For randomization, sealed envelopes were prepared in advance and marked on the inside with a + (RAS treadmill) or - (non_RAS treadmill) by a deputy experimenter (who was not involved in patient management or data analyses). The experimenters who managed the data were blinded to the patients’ allocation.

### Statistical methods

Whether the data were normally distributed, baseline differences, and the homogeneity of variance of the data were assessed using the Shapiro–Wilks and Levene test, respectively. For descriptive purposes, the outcome measures were compared within and between the two groups using the independent sample t-test or Fisher’s test. As we employed an intent-to-treat analysis, we included every subject who was randomized according to a randomized treatment assignment. For the main analysis (gait training-induced changes) of each outcome measure, we employed repeated measures Analysis of Variance (ANOVA) with the factors *group* (two levels) as the dependent variable and *time* (two levels) as the independent variable. The factor *electrode-pair* (6 levels) was added with regard to the EEG data analysis. The reliability intraclass correlation coefficients, their confidence limits and the effect size for clinical outcomes are also provided. Statistical significance was set at *p* < 0.05. *Post-hoc* paired *t-*tests with Bonferroni correction were thus used. A Spearman correlation test was employed to estimate the correlations between significant EEG and gait changes (behavior changes).

## Results

### Baseline

There were no significant clinical-demographic differences between the two groups (25 patients each) at baseline (Tables 1 and 2). Additionally, there were no significant differences in EEG and gait differences between the groups (all *p* > 0.1). Indeed, both groups showed a weak GQI paralleled by weak fronto-centroparietal α/β-ERS during double support in the stance phase, centroparietal α/β-ERD during single support in the stance phase, and frontal β-ERD during single support in the swing phase of the gait cycle. Furthermore, we detected low TRCoh values within the β fronto-centroparietal, β temporal, and α fronto-temporal paths.

**Table 2:** Pre-post clinical parameters. Data are reported as mean ± s.d.

parameter	group	pre	post	*time×group*		*time* F_(1,24)_, p	post-pre *post-hoc*		
				F_(1,48)_, p	ICC(95%cl)		within-group	effect size	between-group
10MWT (sec)	RAS	7.5 ± 5	6.9 ± 5	1,0.7	0.92(0.86 to 0.95)	1, 0.1	0.7	0.06	0.7
	non-RAS	7.4 ± 5	6.7 ± 4			1.3, 0.1	0.6	0.07	
BBS (sec)	RAS	44 ± 8	49 ± 7	1, 0.6	0.08(0.06 to 0.09)	19, < 0.001	< 0.001	0.8	0.5
	non-RAS	44 ± 8	48 ± 9			73, < 0.001	< 0.001	0.8	
FES (scale score)	RAS	34 ± 9	28 ± 9	32, < 0.001	0(−0.28 to 0.28)	45, < 0.001	< 0.001	0.8	< 0.001
	non-RAS	34 ± 9	31 ± 9			1, 0.2	0.2	0.1	
FGA (scale score)	RAS	18 ± 2	22 ± 2	41, < 0.001	0(−0.28 to 0.28)	42, < 0.001	< 0.001	0.8	< 0.001
	non-RAS	17 ± 2	20 ± 2			1, 0.1	0.1	0.1	
TUG (sec)	RAS	11 ± 7	9 ± 9	5, 0.006	0.45(0.2 to 0.65)	43, < 0.001	< 0.001	0.8	0.6
	non-RAS	11 ± 7	10 ± 7			5, 0.04	0.01	0.4	
UPDRS (scale score)	RAS	29 ± 3	21 ± 5	10, < 0.001	0.45(0.2 to 0.65)	16, 0.001	< 0.001	0.8	0.001
	non-RAS	31 ± 5	25 ± 8			9, 0.006	0.006	0.5	

10MWT 10 m walking test, UPDRS Unified Parkinson’s Disease Rating Scale, BBS Berg Balance Scale, FES Tinetti Falls Efficacy Scale, FGA Functional Gait Assessment, TUG timed up-and-go test, RAS Rhythmic Auditory Stimulation, ICC(95%cl) intraclass correlation coefficient and its 95% confidence limits for test-retest reliability calculation

### Clinical outcomes

All patients completed training without reporting any side effects, and none of the patients withdrew from any treatment session, as assessed by the RAS-trained physiotherapists. As we employed an intent-to-treat analysis, we included every subject who was randomized according to the randomized treatment assignment.

ANOVA analysis showed that RAS was superior to non_RAS in improving FES, FGA, TUG, and UPDRS even though a significant time effect of both of the gait trainings was found in all of the outcome measures except 10MWT (Table 2). Specifically, FES improved more in the RAS group than in the non_RAS group (− 18%, *p* < 0.001, and − 9%, *p* = 0.2, respectively; RAS/non_RAS between-group difference + 100%, *p* < 0.001), as did FGA (+ 22%, *p* < 0.001, and + 17%, *p* = 0.1, respectively; RAS/non_RAS between-group difference + 29%, *p* < 0.001), and UPDRS (− 28%, *p* < 0.001, and − 20%, *p* = 0.006, respectively; RAS/non_RAS between-group difference + 40%,*p* = 0.001). However, both groups improved equally in BBS (RAS + 11%, *p* < 0.001, non_RAS + 9%, *p* < 0.001; RAS/non_RAS between-group difference + 22%, *p* = 0.5) and TUG (RAS + 22%, *p* < 0.001, non_RAS + 10%, *p* = 0.01; RAS/non_RAS between-group difference + 20%, *p* = 0.6). Finally, neither group significantly improved on the 10MWT (RAS − 8%, *p* = 0.06, non_RAS − 10%, *p* = 0.07; RAS/non_RAS between-group difference + 20%, *p* = 0.6).

### Kinematic outcomes

The abovementioned clinical changes were paralleled by larger modifications in gait parameters. ANOVA analysis showed that RAS was superior to non_RAS in improving all kinematic measures except for gait cycle duration and speed of gait even though a significant time effect of both of the gait trainings was found in all of the gait parameters (Table 3). Specifically, GQI increased more in the RAS than in the non_RAS training group (RAS + 10%, *p* < 0.001, non_RAS + 4%, *p* = 0.007; RAS/non_RAS between-group difference + 172%, *p* < 0.001) (Table 3). Specifically, GQI changes resulted from the greater reduction observed following RAS than non_RAS in the stance/swing ratio (RAS − 6%, *p* < 0.001, non_RAS − 4%, *p* = 0.009; RAS/non_RAS between-group difference + 43%, *p* < 0.001) and step cadence (RAS − 11%, *p* < 0.001, non_RAS − 5%, *p* = 0.006; RAS/non_RAS between-group difference + 136%, *p* < 0.001) and the increase in stride length (RAS + 35%, *p* = 0.005, non_RAS + 31%, *p* = 0.003; RAS/non_RAS between-group difference + 14%, *p* = 0.01) (Table 3). In contrast, gait cycle duration decreased equally in both groups (RAS − 13%, *p* = 0.002, non_RAS − 9%, *p* = 0.007; RAS/non_RAS between-group difference + 42%, *p* = 0.1), whereas speed of gait increased equally (RAS + 41%, *p* = 0.004, non_RAS + 31%, *p* = 0.006; RAS/non_RAS between-group difference + 31%, *p* = 0.1) (Table 3).

**Table 3:** Statistical data of the effects of gait training on gait kinematic parameters from the baseline (pre) at the end of the rehabilitation period (post)

Parameter	*time×group*		group	pre	post	*time* F_(1,24)_,p	post-pre *post-hoc p*-value		
	F_(1,48)_, p	ICC (95%cl)					within-group	effect size	between-group
GQI (%)	26, < 0.001	0.02 (−0.26 to 0.29)	RAS	80 ± 9	89 ± 10	93, < 0.001	< 0.001	0.8	< 0.001
			non-RAS	81 ± 9	84 ± 9	63, < 0.001	0.007	0.6	
SSR (ratio)	8.7, 0.005	0.05 (−0.22 to 0.33)	RAS	2.1 ± 0.2	2.0 ± 0.2	71, < 0.001	< 0.001	0.5	< 0.001
			non-RAS	2 ± 0.2	1.9 ± 0.2	48, < 0.001	0.009	0.3	
step cadence (Hz)	16, < 0.001	0.03 (−0.25 to 0.3)	RAS	1.56 ± 0.2	1.4 ± 0.2	44, < 0.001	< 0.001	0.8	< 0.001
			non-RAS	1.58 ± 0.2	1.5 ± 0.2	22, < 0.001	0.006	0.4	
stride length (cm)	15, < 0.001	0.03 (−0.25 to 0.31)	RAS	37 ± 4	50 ± 6	22, 0.009	0.005	0.9	0.01
			non-RAS	36 ± 4	47 ± 5	8.2, 0.02	0.003	0.6	
gait cycle duration (sec)	0.2, 0.5	0.07 (0.05 to 0.08)	RAS	2.02 ± 0.2	1.77 ± 0.2	29, < 0.001	0.002	0.8	0.1
			non-RAS	1.96 ± 0.2	1.79 ± 0.2	11, < 0.001	0.007	0.7	
speed of gait (m/s)	0.2, 0.5	0.9 (0.05 to 0.1)	RAS	0.7 ± 0.1	0.9 ± 0.1	20, < 0.001	0.004	0.8	0.1
			non-RAS	0.6 ± 0.1	0.8 ± 0.1	15, < 0.001	0.006	0.8	

*UPDRS* Unified Parkinson’s Disease Rating Scale, *BBS* Berg Balance Scale, *FES* Tinetti Falls Efficacy Scale, *FGA* functional gait assessment, *TUG* timed up-and-go test, *GQI* gait quality index, *SSR* stance-swing ratio, *RAS* rhythmic auditory stimulation, *ICC(95%cl)* intraclass correlation coefficient and its 95% confidence limits for test-retest reliability calculation

### ERD/ERS aftereffects

**Fig. 2:**
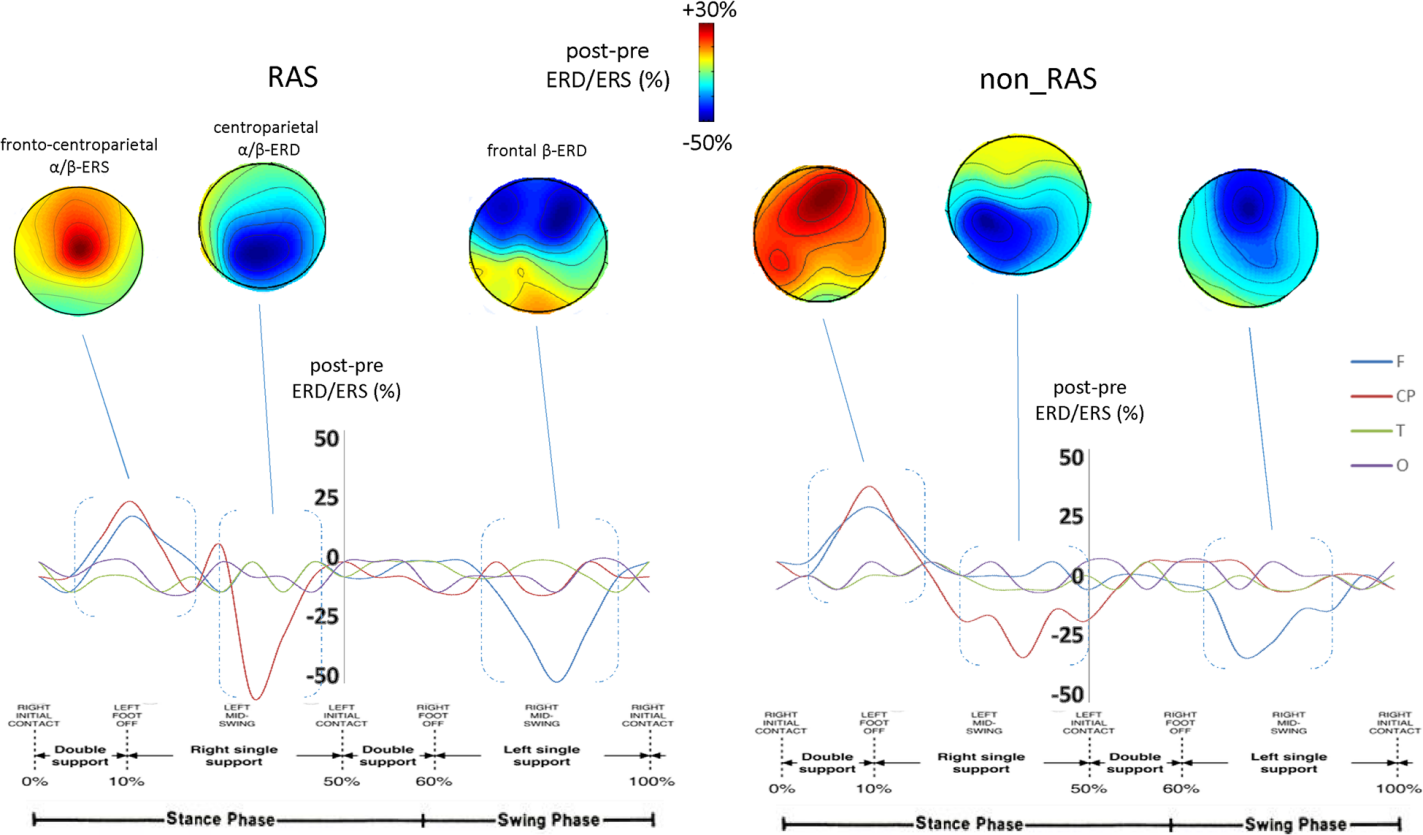
Average post vs. pre changes in ERSs/ERDs and their scalp projections relatively to the full gait cycle in the two groups (RAS and non_RAS gait training). We found a significant strengthening of the central α/β-ERD during single support in the stance phase, of the low frontal β-ERD during the single support in the swing phase, and of the fronto-central α/β-ERS during the double support in the stance phase of the gait cycle. All such changes were more evident following RAS compared to non_RAS training. Average post vs. pre changes in alpha and beta ERD/ERS color maps are coded in blue and red tones, respectively. Electrodes were grouped into frontal F -Fp1/2,F3/4/7/8, centroparietal -C3/4,P3/4-, temporal T -T3/4/5/6, and occipital O -O1/2-

The observed clinical improvement was paralleled by significant changes in gait-related α and β ERS and ERD within the frontal and centroparietal electrodes, which were more evident following RAS than non_RAS training (Fig. 2, Table 4). ANOVA analysis showed that RAS was superior to non_RAS in improving all of the power estimations even though a significant time effect of both of the gait trainings was found in central and frontal α and β ERS and ERD (Table 3). In detail, centroparietal α-ERD during single support in the stance phase increased more in the RAS than in the non_RAS training group (RAS − 32%, *p* < 0.001, non_RAS − 15%, *p* < 0.001; RAS/non_RAS between-group difference + 113%, *p* = 0.01). Similar findings were obtained concerning centroparietal α-ERS during the double support in the stance phase of the gait cycle (RAS + 29%, *p* < 0.001, non_RAS + 10%, *p* < 0.001; RAS/non_RAS between-group difference + 190%, *p* = 0.01), in frontal β-ERD during the single support in the swing phase (RAS − 31%, *p* < 0.001, non_RAS − 12%, *p* < 0.001; RAS/non_RAS between-group difference + 158%, *p* = 0.01), in centroparietal β-ERD during single support in the stance phase (RAS − 29%, *p* < 0.001, non_RAS − 9%, *p* < 0.001; RAS/non_RAS between-group difference + 222%, *p* = 0.01), and in centroparietal β-ERS during double support in the stance phase of the gait cycle (RAS + 31%, *p* < 0.001, non_RAS + 11%, *p* < 0.001; RAS/non_RAS between-group difference + 181%, *p* = 0.01).

**Table 4:** Statistical data of the effects of gait training on EEG findings from the baseline (pre) at the end of the rehabilitation period (post)

parameter		*time×group*		group	post vs. pre % change	*time* F_(1,24)_,p	post-pre *post-hoc p*-values		
		F_(1,48)_, p	ICC (95%cl)				within-group	effect size	between-group
power	CP α-ERD	56, < 0.001	0.01 (−0.27 to 0.28)	RAS	−32	71, < 0.001	< 0.001	0.9	0.01
				non-RAS	−15	33, < 0.001	< 0.001	0.7	
	CP α-ERS	20, < 0.001	0.02 (−0.25 to 0.30)	RAS	+ 29	53, < 0.001	< 0.001	0.9	0.01
				non-RAS	+ 10	36, < 0.001	< 0.001	0.7	
	F β-ERD	89, < 0.001	0.01 (−0.27 to 0.28)	RAS	−31	76, < 0.001	< 0.001	0.9	0.01
				non-RAS	−12	59, < 0.001	< 0.001	0.7	
	CP β-ERD	92, < 0.001	0.01 (−0.27 to 0.28)	RAS	−29	30 < 0.001 22, < 0.001	< 0.001	0.8	0.01
				non-RAS	−9		< 0.001	0.5	
	CP β-ERS	73, < 0.001	0.01 (−0.27 to 0.28)	RAS	+ 31	39, < 0.001	< 0.001	0.9	0.01
				non-RAS	+ 11	29, < 0.001	< 0.001	0.7	
TRCoh	β F-CP	10, < 0.001	0.05 (−0.23 to 0.32)	RAS	+ 63	18, 0.001	< 0.001	0.8	0.01
				non-RAS	+ 21	9, 0.001	0.001	0.6	
	β F-T	4.7, 0.04	0.10 (−0.18 to 0.36)	RAS	+ 43	25, < 0.001	< 0.001	0.8	0.01
				non-RAS	+ 21	17, < 0.001	0.001	0.7	
	α F-T	6.3, 0.01	0.07 (−0.21 to 0.34)	RAS	+ 36	15, 0.002	0.001	0.8	< 0.001
				non-RAS	+ 18	2.3, 0.1	0.1	0.2	

*ERD* event-related desynchronization, *ERS* event-related synchronization, *TRCoh* task-related coherence, *RAS* rhythmic auditory stimulation, *ICC(95%cl)* intraclass correlation coefficient and its 95% confidence limits for test-retest reliability calculation, *F* frontal, *CP* centroparietal, *T* temporal

### TRCoh aftereffects

ANOVA analysis showed that RAS was superior to non_RAS in improving all of the TRCoh estimations, even though a significant time effect of both of the gait trainings was found in β fronto-centroparietal and fronto-temporal TRCoh (but in α fronto-temporal only following non_RAS) (Table 4). Specifically, fronto-centroparietal β-TRCoh increased more in the RAS than in the non_RAS training group (RAS + 63%, *p* < 0.001, non_RAS + 21%, *p* = 0.001; RAS/non_RAS between-group difference + 200%, *p* = 0.01). Similar findings were obtained concerning fronto-temporal β-TRCoh (RAS + 43%, *p* < 0.001, non_RAS + 21%, *p* = 0.001; RAS/non_RAS between-group difference + 104%, *p* = 0.01). However, fronto-temporal α-TRCoh increased only following RAS training (RAS + 36%, *p* < 0.001, non_RAS + 18%, *p* = 0.1; RAS/non_RAS between-group difference + 100%, *p* < 0.001) (Table 4).

### Clinical-electrophysiological correlations

**Fig. 3:**
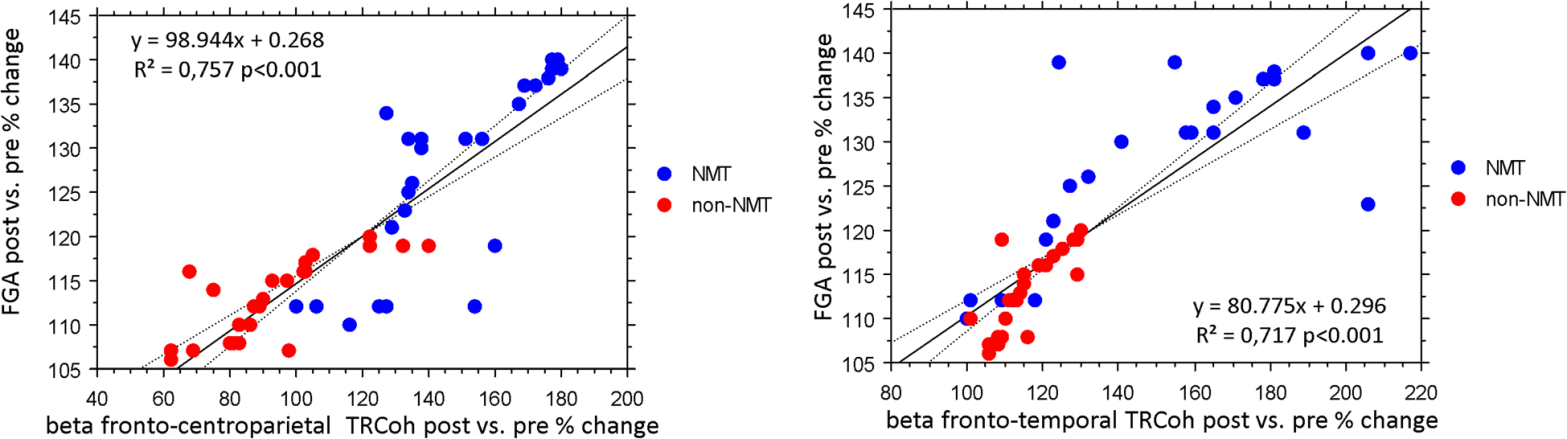
Scatter plot graphs for the relationship between the clinical and neurophysiological data

A Spearman test showed that there were significant correlations between FGA improvement (primary outcome) and the increases in fronto-centroparietal (*r* = 0.757, *p* < 0.001) and fronto-temporal beta range connectivity increase (*r* = 0.717, *p* < 0.001) (Fig. 3).

## Discussion

Our data indicate that RAS training offers additional advantages in terms of overall gait quality, balance, number and length of strides compared to non_RAS, as reported in the literature. This finding is important from a rehabilitative perspective, given that poor gait in patients with PD is characterized by an increase in the number of steps [14, 20, 69, 77]. On the other hand, RAS training was not superior to non_RAS concerning the improvement in gait speed, turning, and stride duration, as formerly reported (Miller et al. 1996; [14, 20, 43, 69, 77];), thus suggesting that these improvements were influenced by the rehabilitative program itself rather than cueing. However, improving the speed of gait and turning is an important target in PD rehabilitation [14, 20, 69, 77]. Therefore, this finding also represents an important rehabilitative endpoint [14, 20, 69, 77].

The novelty of our study is that we reveal a putative neurophysiological mechanisms explaining the greater strength of RAS training (by using GaitTrainer3) when obtaining clinical improvement compared to an equivalent dose of non_RAS training using EEG.

Some previous EEG studies have characterized cortical oscillations related to gait in patients with PD, and a few have provided robust data on EEG power; however, even fewer have explored functional connectivity [78]. Overall, there is some evidence showing that cortical activity abnormally increases during gait in patients with PD, and this likely represents a cortical compensation phenomenon reflecting subcortical (basal ganglia and the cerebellum) dysfunction [78]. Unfortunately, there is a paucity of correlation analyses of cortical and behavioral outcomes, and these have mainly performed functional imaging. In particular, it has been shown that gait impairment is correlated with the deterioration of a fronto-centroparietal network, beyond BG, the level of cortical activity, the increased activity of the prefrontal cortex, and the cortical timing metrics [22, 30–32].

However, more data are available from functional neuroimaging studies than those using EEG approaches [4, 37, 78], whereas there is no significant EEG data related to RAS gait training aftereffects. We found that the RAS training-induced gait improvement depended on stronger entrainment of fronto-centroparietal and fronto-temporal electrode connectivity than was required by non_RAS training, as suggested by the significant correlation between the changes in connectivity measures and the behavior (gait) indices and the greater modulation of frontal and centroparietal α and β power related to specific parts of the gait cycle.

The changes in beta range connectivity that occurred as part of RAS training were the most important contributors to the observed clinical improvement (as per clinical-behavior correlation analysis) and are likely to depend on associative plasticity between the acoustic cues coupled to walking ([4, 26, 56, 81, 82, 92];). Hence, the external pacing cues used in treadmill walking may interact with the mechanical pacing of footfalls on the running belt. Indeed, patients had to walk while synchronizing their footsteps to the salient beats of the music, thus leading to audiomotor integration phenomena mediated through fronto-temporal and fronto-centroparietal pathways ( [26, 81, 82]; Yeterian and Pandya, 1998 [4, 56];). This likely allowed the generation of a more physiological and rhythmic gait by integrating implicit and explicit timing mechanisms to compensate for the internal pacing deterioration [61]. Moreover, the external cueing modality we adopted harnessed implicit timing, which is mostly intact in PD, thus still allowing automatic timing [23]. Finally, the greater fronto-temporal connectivity observed following RAS than non_RAS is likely to depend on the modulation of β-oscillations among a wide network of auditory, motor, and associative cortices by part of auditory cueing, thus promoting motor activation patterns [4, 23]. Sensorimotor rhythms are finely tuned during gait training and represent timely selective top-down control from the cortex to subcortical structure (and then to the muscles), and they thus serve as a strong promoter of the motoric status quo and controller of gait stability and adaptations, sensory processing of the lower limbs, visuomotor integration, and speed, depending on the current motor scenario [34, 45]. It has been reported that the spatiotemporal extent of alpha and beta synchronization within fronto-centroparietal and fronto-temporal electrodes is inappropriately increased and its reactivity diminished in PD, mainly owing to BG impairment, reduced dopamine release, and intrinsic cortical excitability abnormalities [23, 24, 34, 36, 45, 60]. This rhythm deterioration is strongly correlated with the clinical picture at baseline and reflects the inability of patients with PD to modulate their gait cycle according to walking necessities and given the clinical improvement observed following levodopa treatment and deep brain stimulation (DBS) reported in the data of the literature [34, 45]. Therefore, the strong spatiotemporal changes in sensorimotor rhythms observed across the gait cycle (and thus the clinical improvement) obtained by coupling music and gait training may depend on the precise modulation of dopamine release by internal and external timing mechanisms that are triggered by music as these allow the fine-tuning of gait cycle parameters according to the motor scenario and motor task demand in a way resembling levodopa and DBS [34, 36, 45]. However, we can only speculate on the neurophysiological similarities of the effects of music, levodopa, and DBS as the first is less discriminating when focusing on the particularly extensive beta synchrony, leaving undisturbed the other periods compared to levodopa and DBS [34, 45]. Nonetheless, it has been reported in healthy participants that the presentation of RAS significantly improved finger tapping task performance, leading to significantly reduced DA responses in the left ventral striatum [36]. Thus, the potential role of RAS in modulating DA responses should be confirmed in PD patients, considering the dopaminergic role in the enhancement of motor control in PD with the consequent implications in neurorehabilitation.

It has been proposed that cerebello-thalamo-cortical motor networks could compensate for the detrimental BG-thalamo-cortical motor network functions related to internal timing processing [16, 63, 74, 87]. Indeed, there is evidence that temporal rhythmic auditory information may assist compensatory mechanisms through network-level effects, reflected in increased interaction between auditory and executive networks that in turn modulate activity in cortico-cerebellar networks [4]. We hypothesized that the cerebellum contributes to mediate more of the fronto-centroparietal and fronto-temporal temporal electrode connectivity (and the clinical-kinematic improvement) following RAS training then non_RAS training. It has been shown that rhythmic cerebellar stimulation by means of oscillatory transcranial currents delivered at frequencies resembling an intrinsic musical tempo largely shapes fronto-parietal connectivity and the sensorimotor rhythms related to the fine regulation of gait parameters [57, 58]. Therefore, it is likely that the cerebellum contributes to internal timing mechanisms when properly stimulated by rhythmic external cues, or at least acoustic cues. Nonetheless, the involvement of the cerebellum by part of the RAS needs to be further studied to better characterize the neurophysiological basis, including which cue typology is required and the rhythm specificity. In fact, over-activation of the cerebellum may worsen gait as suggested by studies of non-invasive cerebellar stimulation in PD. [53]

Another main finding of our study is that α frequency range fronto-temporal connectivity was only involved in the RAS group. This functional connectivity is strongly linked to cognitive performance in PD as it deteriorates in parallel with cognitive decline [34]. Moreover, the potentiation of frontotemporal connectivity is also important in motor and cognitive rehabilitation [22]. Given that α deterioration is a marker of the degeneration of the ascending diffuse projection systems that control attention [34], a key advantage of using music as an external cue is that it increases the attention level, as reflected by the low variability of the outcome measures following RAS and the consequential improvement in patient participation and performance.

### Limitations

The main limitation of our study is the lack of a follow-up period. However, it has been shown that patients with PD who are provided with cued gait training do not retain the obtained clinical improvement after 3 months [13]. This probably depends on the progression of neurodegeneration and the detrimental implicit learning in patients with PD. It is likely that retention could be promoted by long-term, less intensive, home rehabilitation. Therefore, future investigations are needed to verify this issue. Further, future directions should include an examination of the EEG changes that occur during over-ground walking and not just walking on a treadmill. Moreover, our time-frequency analysis focused only on the alpha and beta frequency bands. The roles of the other frequency bands deserve further investigation.

Another limitation is that the patients received extended daily rehabilitation training for 8 weeks. Therefore, the changes may not reflect only the differences between RAS and non-RAS training. Even this issue deserves further investigation with different control groups.

We found declines in fronto-parietal connectivity at baseline, whereas it was previously reported that patients with PD show an increase in cortico-cortical functional connectivity (this may reflect a compensatory mechanism to overcome motor-cognitive limitations). However, this over-connectivity was found to be limited to the early stages of the disease, whereas our patients had a disease duration of approximately 10 years.

Finally, it would be interesting to test whether the effect of RAS on patients’ gait parameters depends on whether the music was or was not appreciated by the patient, in comparison to the effect of a musical piece that was chosen at the same for all patients.

## Conclusion

Our data suggest that RAS may be a useful, add-on, gait rehabilitation strategy in PD as auditory cueing can specifically target motor cortical beta frequency range synchrony during steady-state treadmill walking in patients with PD. This modulation sustained greater clinical improvement following RAS gait training than non_RAS gait training. This extensive oscillatory recruitment may represent a bypass of the damaged circuitry of internal pacing by part of a broader network encompassing the cerebellum and different cortical areas. Therefore, the brain could recalibrate its internal pacing mechanisms by harnessing the rich sensorimotor feedback signals provided by the music-gait coupling.

Obtaining a better understanding of the neurophysiological mechanisms underlying the cortical control of cued gait in patients with PD may provide us with information that would allow us to design interventions targeting such cortical mechanisms using, e.g., transcranial magnetic stimulation, transcranial alternating current stimulation or, as in our study, cueing strategies. Targeting the functional connectivities along fronto-centroparietal/temporal electrodes and the α and β rhythms related to specific parts of the gait cycle may be an important issue in the motor rehabilitation of patients with PD when aiming to mitigate walking disturbances in these patients. In other words, identifying the neurophysiological mechanisms underlying RAS-induced gait improvement may help clinicians to develop patient-tailored rehabilitative approaches based on the selective impact of cues on gait parameters, thus making gait training highly individualized and optimizing its efficacy.

## Abbreviations

10MWT: 10-Meter Walking Test
ANOVA: Analysis of Variance
BBS: Berg Balance Scale
BG: Basal ganglia
DBS: Deep brain stimulation
EEG: Electroencephalography
ERD: Event-related desynchronization
ERS: Event-related synchronization
FES: Tinetti Falls Efficacy Scale
FGA: Functional Gait Assessment
FIR: Finite impulse response
GQI: Gait quality index
HS: Heel strike
ICA: Independent Component Analysis
MCID: Minimal clinically important difference
PD: Parkinson’s disease
RAS: Rhythmic auditory stimulation
TRCoh: Task-related coherence
TUG: Timed Up-and-Go test
UPDRS: Unified Parkinson Disease Rating Scale
